# Localization of TSH-secreting pituitary adenoma using 11C-methionine image subtraction

**DOI:** 10.1186/s13550-022-00899-7

**Published:** 2022-05-07

**Authors:** Daniel Gillett, Russell Senanayake, James MacFarlane, Merel van der Meulen, Olympia Koulouri, Andrew S. Powlson, Rosy Crawford, Bethany Gillett, Nick Bird, Sarah Heard, Angelos Kolias, Richard Mannion, Luigi Aloj, Iosif A. Mendichovszky, Heok Cheow, Waiel A. Bashari, Mark Gurnell

**Affiliations:** 1 Department of Nuclear Medicine, Cambridge University Hospitals NHS Foundation Trust, Cambridge Biomedical Campus, Hills Road, Cambridge, CB2 0QQ UK; 2 Cambridge Endocrine Molecular Imaging Group, University of Cambridge, Addenbrooke’s Hospital, Cambridge Biomedical Campus, Hills Road, Cambridge, CB2 0QQ UK; 3 East Anglian Regional Radiation Protection Service, Cambridge University Hospitals NHS Foundation Trust, Cambridge Biomedical Campus, Hills Road, Cambridge, CB2 0QQ UK; 4 Division of Neurosurgery, Department of Clinical Neurosciences, University of Cambridge & Addenbrooke’s Hospital, Cambridge, CB2 0QQ UK; 5 Department of Radiology, University of Cambridge, Cambridge Biomedical Campus, Hills Road, Cambridge, CB2 0QQ UK; 6 Metabolic Research Laboratories, Wellcome-MRC Institute of Metabolic Science University of Cambridge, National Institute for Health Research Cambridge Biomedical Research Centre, Addenbrooke’s Hospital, Hills Road, Cambridge, CB2 0QQ UK

**Keywords:** ^11^C-methionine PET, Human pituitary tumors, Normalization, Subtraction imaging

## Abstract

**Background:**

Pituitary adenomas (PA) affect ~ 1:1200 of the population and can cause a wide range of symptoms due to hormone over-secretion, loss of normal pituitary gland function and/or compression of visual pathways, resulting in significantly impaired quality of life. Surgery is potentially curative if the location of the adenoma can be determined. However, standard structural (anatomical) imaging, in the form of MRI, is unable to locate all tumors, especially microadenomas (< 1 cm diameter). In such cases, functional imaging [^11^C-methionine PET/CT (Met-PET)] can facilitate tumor detection, although may be inconclusive when the adenoma is less metabolically active. We, therefore, explored whether subtraction imaging, comparing findings between two Met-PET scans with medical therapy-induced suppression of tumor activity in the intervening period, could increase confidence in adenoma localization. In addition, we assessed whether normalization to a reference region improved consistency of pituitary gland signal in healthy volunteers who underwent two Met-PET scans without medical suppression.

**Results:**

We found that the mean percentage differences in maximum pituitary uptake between two Met-PET scans in healthy volunteers were 2.4% for (SUVr) [cerebellum], 8.8% for SUVr [pons], 5.2% for SUVr [gray matter] and 23.1% for the SUVbw [no region]. Laterality, as measured by contrast–noise ratio (CNR), indicated the correct location of the adenoma in all three image types with mean CNR values of 6.2, 8.1 and 11.1 for SUVbw, SUVbwSub and SUVrSub [cerebellum], respectively. Subtraction imaging improved CNR in 60% and 100% of patients when using images generated from SUVbw [no region] and SUVr [cerebellum] scans compared to standard clinical SUVbw imaging.

**Conclusions:**

Met-PET scans should be normalized to the cerebellum to minimize the effects of physiological variation in pituitary gland uptake of 11C-methionine, especially when comparing serial imaging. Subtraction imaging following endocrine suppression of tumor function improved lateralization of PA when compared with single time point clinical Met-PET but, importantly, only if the images were normalized to the cerebellum prior to subtraction.

## Background

Although the majority of pituitary tumors are benign (pituitary adenomas, PA), they can be associated with significant morbidity and increased mortality and affect approximately 1:1200 of the general population [1]. Symptoms and signs may be due to compression of adjacent anatomical structures (e.g., superior extension compressing the optic chiasm with resultant visual loss) and syndromes associated with hormone excess [e.g., growth hormone (GH) in acromegaly; ACTH in Cushing Disease; thyroid-stimulating hormone (TSH) in thyrotropinomas (TSHomas)] or insufficiency due to failure of the normal pituitary gland (hypopituitarism) [2]. It is not surprising then that many patients with pituitary tumors report significantly impaired quality of life, even worse than for some cancers [3, 4]. Medical treatment can be very effective in certain PA subtypes (e.g., dopamine agonist for prolactinomas), but surgery remains the preferred management option for many patients (e.g., acromegaly, Cushing Disease, thyrotropinoma), aiming to preserve/restore vision and/or permanently remove the source of hormone excess [5–7]. However, transsphenoidal pituitary surgery (TSS) is challenging due to the close proximity of critical structures (e.g., internal carotid artery within the cavernous sinus) to the normal gland, which may limit the scope for surgical resection. Conventionally, gadolinium-enhanced T1- and T2-weighted MRI is used for preoperative localization of the tumor and to inform surgical planning. However, anatomical information obtained on conventional imaging does not always locate the tumor, especially in the case of pituitary microadenomas (< 10 mm diameter) or when image contrast is low. Recently, we and others have demonstrated the potential clinical utility of molecular imaging to address this challenge [8–10]. Specifically, ^11^C-methionine PET (Met-PET) has been shown to aid localization of newly diagnosed and recurrent tumors across the spectrum of PA subtypes [9, 10].

However, there remain a subgroup of patients in whom even the combination of MR and Met-PET yield equivocal findings. In this setting, the patient and clinician are faced with a difficult choice: Should they proceed to surgery with the likelihood that a more extensive exploration of the whole gland will be required in an attempt to localize the tumor—this approach is reasonable but may not achieve surgical cure if the tumor cannot be identified and carries a risk of damage to adjacent critical structures (e.g., carotid artery and normal pituitary gland). Alternatively, drug treatment can be deployed as primary therapy, but may be associated with side effects and is often required lifelong with attendant significant costs [e.g., > $100 K per annum for somatostatin receptor ligand (SRL) therapy] [11]. Such treatment is often very effective. For example, TSHomas demonstrate somatostatin receptor (SSTR, predominately SSTR2 and SSTR5) expression (12). Importantly, in a single illustrative case, suppression of the activity of the TSHoma (as confirmed by correction of the hyperthyroidism clinically and biochemically) appeared to correlate with reduction/extinction of the signal seen on Met-PET imaging [9]. We therefore hypothesized that using a subtraction technique similar to that routinely used in SPECT for parathyroid adenoma [13] and ictal SPECT imaging [14–17] could aid pituitary tumor localization using Met-PET. These techniques use normalization to prepare the images for subtraction by either scaling the images, so the mean intensity in the two datasets is matched, or by scaling the image intensity using a reference region to create an image of ratios (known as standardized uptake value ratios—SUVrs) [18]. Ictal subtraction imaging has also been performed using a combination of SPECT and PET [19], while a novel application of PET subtraction imaging has successfully identified sepsis in multiple sites using 18F-FDG imaging before and after antimicrobial therapy in a patient suffering recurrent sepsis episodes [16]. These subtraction techniques increase the contrast-to-noise ratio by reducing the surrounding signal that is consistent between scans while highlighting those areas that demonstrate change.

The subtraction imaging techniques described above differ in their approach; subtraction imaging to detect sepsis uses standardized uptake value—body weight (SUVbw) images, whereas parathyroid and ictal subtraction imaging uses normalized images. Here, we have applied both techniques and assessed their effectiveness to aid pituitary tumor localization compared to standard Met-PET. To effectively normalize the images, an appropriate reference region must be selected. Therefore, we compared three commonly used reference regions from other nuclear medicine studies of the central nervous system (cerebellum, pons and gray matter [18, 20, 21]) to determine which would yield the most consistent tracer uptake and should be used to normalize the datasets.

## Methods

### Population groups

Two groups of participants were included: six healthy volunteers and ten patients with a thyrotropinoma (subsequently confirmed histologically following pituitary surgery). The healthy volunteers were control subjects in a study which included an assessment of the reproducibility of Met-PET findings on scans performed at a one-month interval (Clinical Trial ID: *Research Registry* 2070). Data from healthy volunteers allowed the most appropriate reference region for normalization of the pituitary uptake to be determined. The patient group was used to explore which subtraction technique provided the clearest localization of the pituitary tumor (matched with findings at surgery). The cohort of patients with TSH-secreting pituitary adenomas included 2 men and 8 women, with a mean age of 58 years (range 27–75). In each case, the patient had confirmed hyperthyroxinemia (raised free thyroid hormone levels) with non-suppressed TSH and met clinical and laboratory criteria for a diagnosis of autonomous tumoral TSH secretion [22]. All patients had microadenomas as reported by consultant neuroradiologists with expertise in pituitary disease and were subsequently reviewed at the regional pituitary multi-disciplinary team meeting. In four patients, these were visualized on standard clinical MRI [Spin Echo T1-weighted (T1SE) pre- and post-gadolinium and Fast T2-weighted SE (T2) FSE] and ranged from 6 to 10 mm in maximum diameter. A further three microadenomas were visualized on gradient recalled echo MRI (GRE, i.e., volumetric sequences used for PET to MRI registration) and ranged from 3 to 4 mm in maximum diameter. In the other three patients, the location of the microadenoma (4–5 mm in maximum diameter) could only be appreciated on GRE MRI when subsequently identified on Met-PET. Met-PET identified a clear adenoma in five patients, with suspected adenomas in a further three patients, but no definite abnormality in the remaining two cases. Each patient was treated with depot first generation somatostatin receptor ligand (SRL) (Lanreotide Autogel®, 90 mg 4-weekly for 3 doses) as per standard clinical practice in preparation for surgery (to alleviate symptoms and mitigate the perioperative risks associated with uncontrolled thyrotoxicosis) [22]. In eight patients, the suspected site of a TSH-secreting microadenoma as visualized on MRI and PET was confirmed on histology following transsphenoidal surgery. Two patients elected to continue with primary somatostatin receptor ligand therapy and both achieved sustained normalization of free thyroid hormone and TSH levels consistent with a tumoral origin of autonomous TSH secretion [23, 24]. These subjects were scanned according to clinical protocols prior to and following SRL therapy, which was anticipated to suppress Met-PET uptake in the tumor. This suppression of tumor function was confirmed clinically (resolution of symptoms) and biochemically (with normalization of thyroid hormone levels). Measurement of other pituitary hormones confirmed no other significant change in pituitary gland status.

### Imaging procedure

Imaging was performed using a Discovery 690 PET/CT scanner (GE Healthcare, Chicago, Illinois, USA) 20 min (range 19–21 min) after administration of 382 MBq (range 293–411 MBq) of ^11^C-Methionine. One 15 cm bed position centered on the subject’s pituitary gland was acquired for 20 min. The images were reconstructed with OSEM iterative reconstruction using 3 iterations and 24 subsets, 128 × 128 matrix size, 2 mm Gaussian filter, scatter correction, CT-measured attenuation correction, time of flight and point spread function correction (SharpIR, GE). An unenhanced CT scan was acquired with 140 kV, fixed mA of 220, a rotation speed of 0.5 s, a pitch of 0984:1, 30 cm field of view, a slice thickness of 1.25 mm and a 1.25 mm spacing interval and reconstructed using filtered back projection.

### MR imaging

MRI was performed with either a GE Optima™ MR450w 1.5 Tesla scanner (GE Healthcare, Chicago, Illinois, USA) or a GE Signa™ 3.0 Tesla scanner (GE Healthcare, Chicago, Illinois, USA) with a head coil using a fast spoiled gradient (recalled) echo (FSPGR) sequence. The sequence parameters were repetition time (TR) 11.5 ms, echo time (TE) 4.2 ms, slice thickness 1 mm and 256 × 256 matrix with 1 mm × 1 mm pixels. The images were acquired after a contrast injection of 0.1 mmol/kg gadobutrol (Gad).

### Image registration

**Fig. 1:**
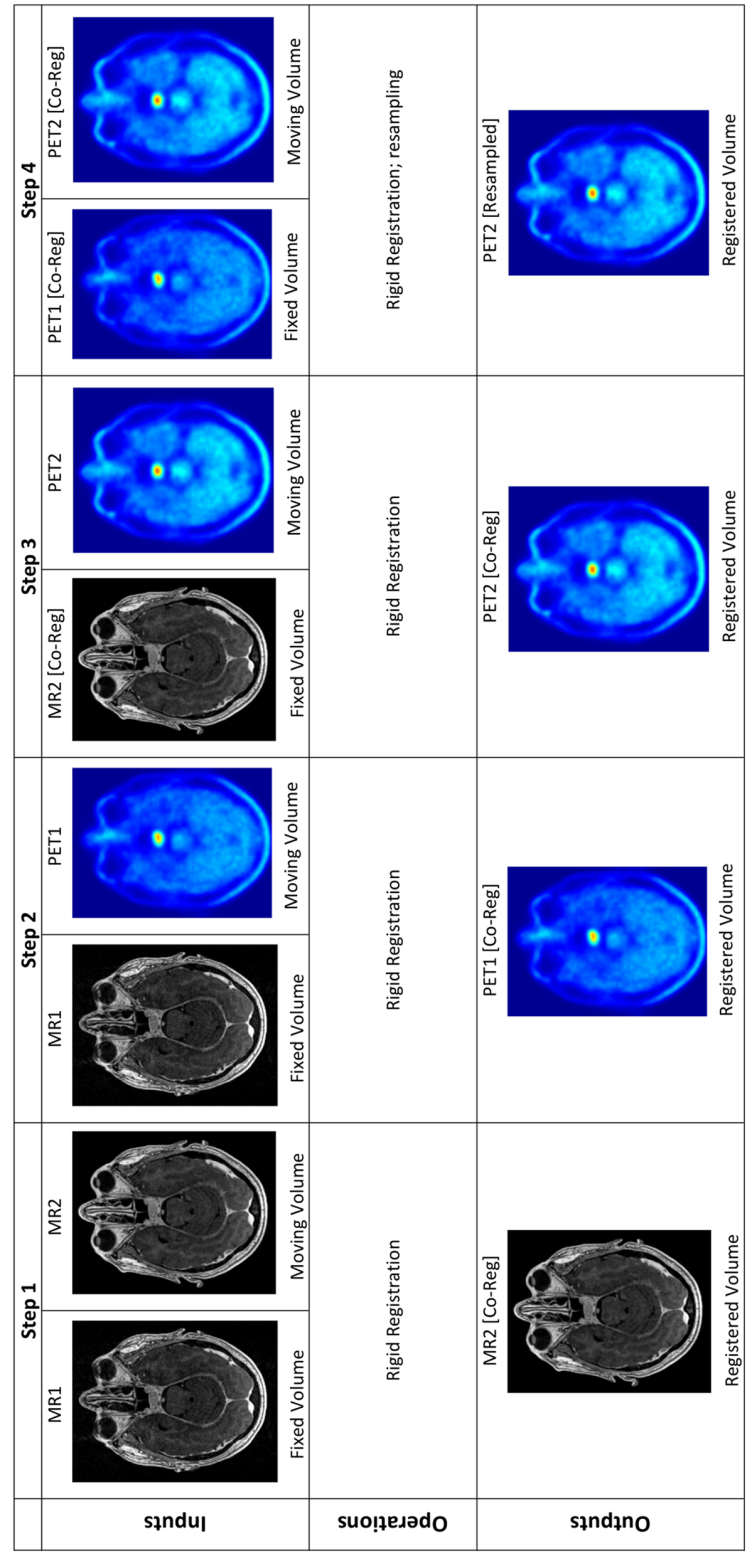
Registration steps. Step 1: MR2 is registered to MR1 using rigid registration. Step 2: PET1 is registered to MR1 using rigid registration. Step 3: PET2 is registered to MR2 [Co-Reg]. Step 4: PET2 [Co-Reg] is registered to PET1 [Co-Reg] and resampled into a matrix of the same size and shape

All registrations were performed in 3D Slicer [25] (version 4.10.2, 05–2019) using multi-modality rigid registration with six degrees of freedom, a maximum number of iterations of 1000 and a sampling ratio of 0.1%. To generate the required co-registered baseline and on-suppression Met-PET/MR images, the following steps were used (illustrated in Fig. 1).

1. The on-suppression MR (MR2) was registered to the baseline MR (MR1) resulting in MR2 [Co-Reg].
2. The baseline Met-PET (PET1) was registered to the baseline MR (MR1) to create PET1 [Co-Reg].
3. The on-suppression PET (PET2) was registered to the on-suppression MR (MR2 [Co-Reg]) resulting in PET2 [Co-Reg].
4. The PET2 [Co-Reg] was resampled into a matrix of the same size and shape as PET1 [Co-Reg] to create PET2 [Resampled].

Step 4 was required to enable voxel-wise manipulations to produce the subtraction images without the requirement for position and interpolation calculations while using the world coordinate system.

### PET normalization

**Fig. 2:**
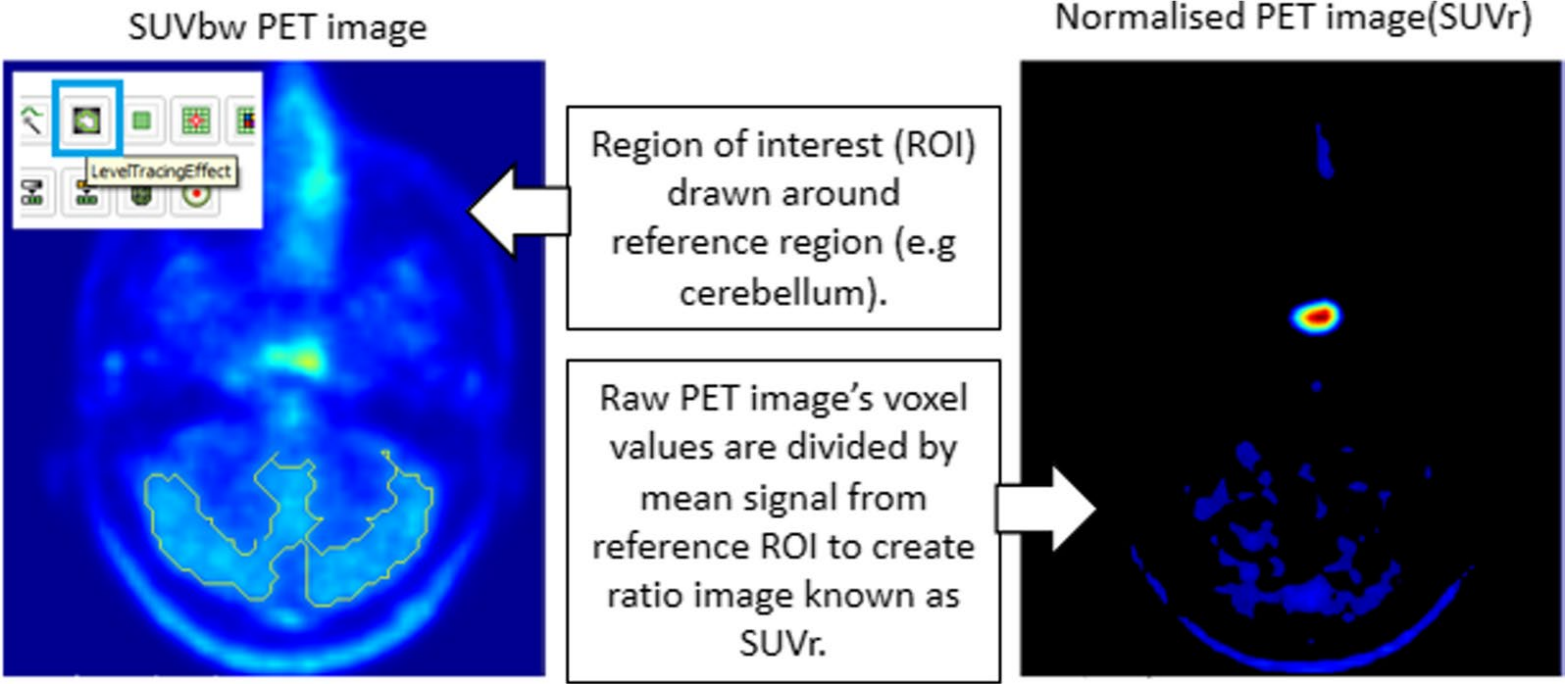
Normalization of PET images. ROI drawn around the reference region (cerebellum shown here) on one slice of the PET image. The PET images are divided by the mean signal from the reference ROI to create a ratio image known as standardized uptake value ratios (SUVrs)

Normalized PET images (SUVr) were created by dividing the voxel values of the PET image by the mean voxel value in a reference region (Fig. 2). This process was implemented in a scripted 3D Slicer module (Fig. 2). The regions were drawn using a local thresholding tool on one or two representative slices of the PET images. The location of this reference region was optimized by finding which of the potential reference regions used for normalization (cerebellum, pons and gray matter) gave the most consistent pituitary tracer uptake in scans from healthy volunteers imaged one month apart. Once the images had been normalized to the reference regions, consistency was measured by finding the absolute percentage difference between the maximum signal in the pituitary gland at both time points. The region with the lowest mean absolute difference was considered the most appropriate region for normalization.

### Subtraction images

**Fig. 3:**
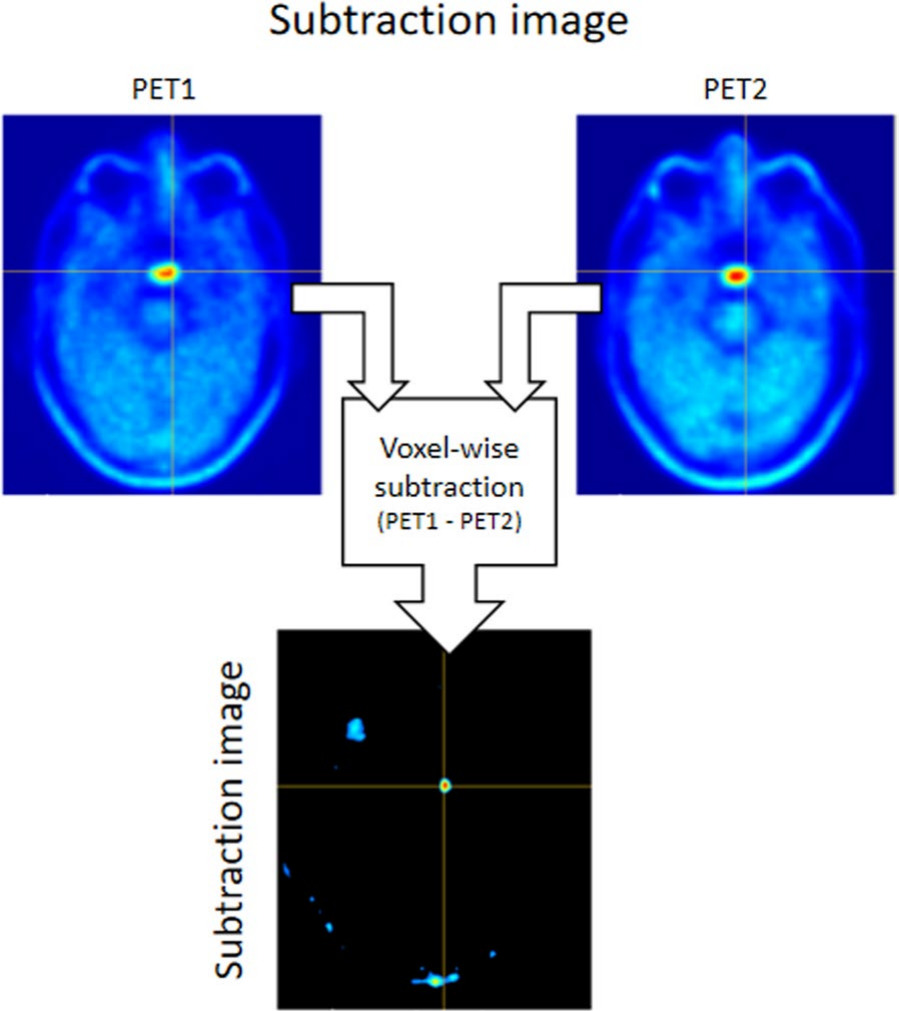
Subtraction imaging techniques. Subtraction images are created by performing a voxel-wise subtraction of the second (suppressed) Met-PET scan from the first baseline Met-PET scan

Subtraction images were created by subtracting the on-suppression Met-PET image (PET2[Resampled]) from the baseline Met-PET image (PET1[Co-Reg]) (Fig. 3). This process was performed using SUVbw Met-PET images and the normalized SUVr (using the optimal reference region) Met-PET images to create two subtraction images for comparison (known as SUVbwSub and SUVrSub, respectively). This process was scripted using 3D Slicer’s native Python environment. The entire process, including registration, was completed in less than 5 min.

### Assessment methodology

**Fig. 4:**
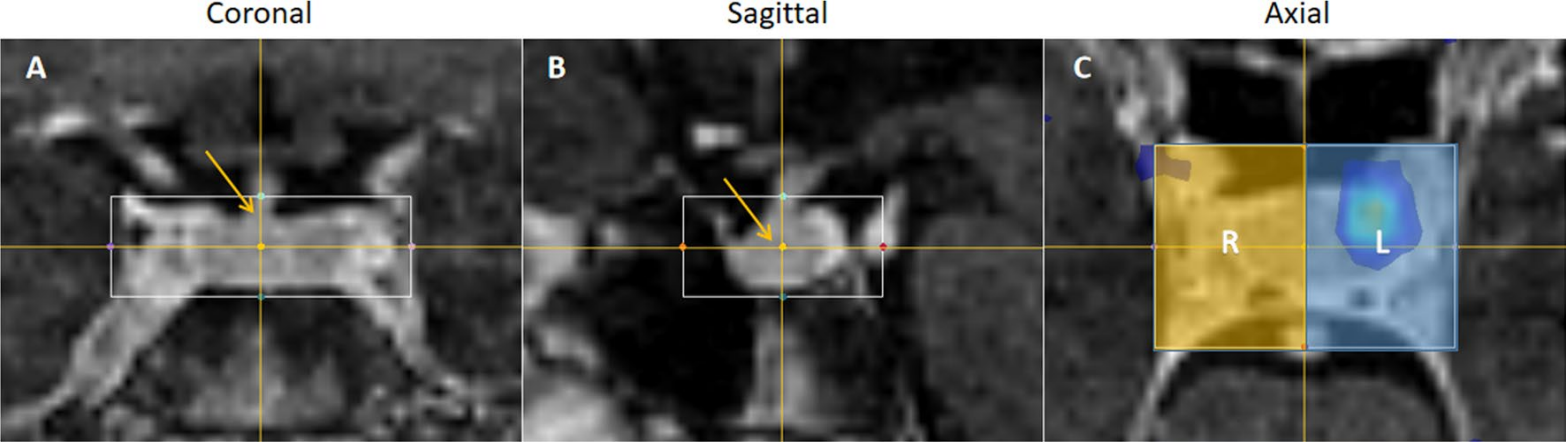
Lateralization CNR.** a** Coronal plane of FSPGR MR image used to find the midline of pituitary stalk. **b** Sagittal plane used to find the center of the pituitary gland. **c** Illustration of volumes of interest used to find left [blue] and right [yellow] maximum signals

To quantitatively compare subtraction images using SUVbw images and SUVr images, ten patients who had confirmed lateralization of a microadenoma (< 10 mm) were assessed using a semi-automated technique. This was undertaken by assessing lateralization using a modified contrast-to-noise ratio equation which selected the maximum signal from each side of the pituitary gland. The midline of the gland was defined on the FSPGR MR sequences as the point of insertion of the infundibulum (pituitary stalk) viewed in the coronal plane (Fig. 4a). Thereafter, using the sagittal plane (so that the midline position could not be altered), the center of the gland was located (Fig. 4b). A rectangular volume of interest was centered at this point and was used to bisect the gland (Fig. 4c). The maximum value in each side of the gland was found, and the contrast between these maximum values was taken as a measure of lateralization. This contrast was then divided by the noise (standard deviation in the normalization ROI) to give a contrast-to-noise ratio (CNR) (see Eq. 1).

1 $${\text{CNR}} = ({\text{Max}}\;{\text{Signal}} - {\text{Max }}\;{\text{Contralateral}}\;{\text{ Signal}})/{\text{Standard}}\;{\text{ Deviation}}\;{\text{ in}}\;{\text{ reference }}\;{\text{ROI}}$$

where Max Signal can be either in the left or the right side of the pituitary gland (relative to the pituitary stalk) and the Max Contralateral Signal is from the opposite side of the gland to the Max Signal.

The mean CNR for both techniques was found, and the technique with the highest value was considered the most appropriate for highlighting the differences.

## Results

### Normalization

**Fig. 5:**
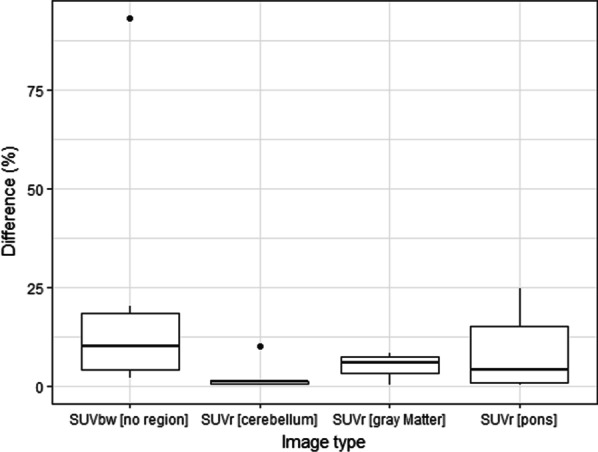
Box plots of maximum signal differences. Absolute percentage differences of the maximum signal in the pituitary gland, comparing pairs of images from healthy volunteers normalized to different regions

To determine the reference region to use for normalization, the mean absolute percentage difference between the maximum signal in the pituitary gland at both time points was found using three reference regions (cerebellum, gray matter and pons) and using SUVbw without normalization. The mean absolute percentage differences were found to be 2.4% for SUVr [cerebellum], 8.8% for SUVr [pons], 5.2% for SUVr [gray matter] and 23.1% for the SUVbw [no region]. The percentage differences are shown as a box plot in Fig. 5. Based on these results, the cerebellum was chosen as the reference region for normalization in the comparison of the subtraction imaging and will be known as SUVrSub [cerebellum].

**Fig. 6:**
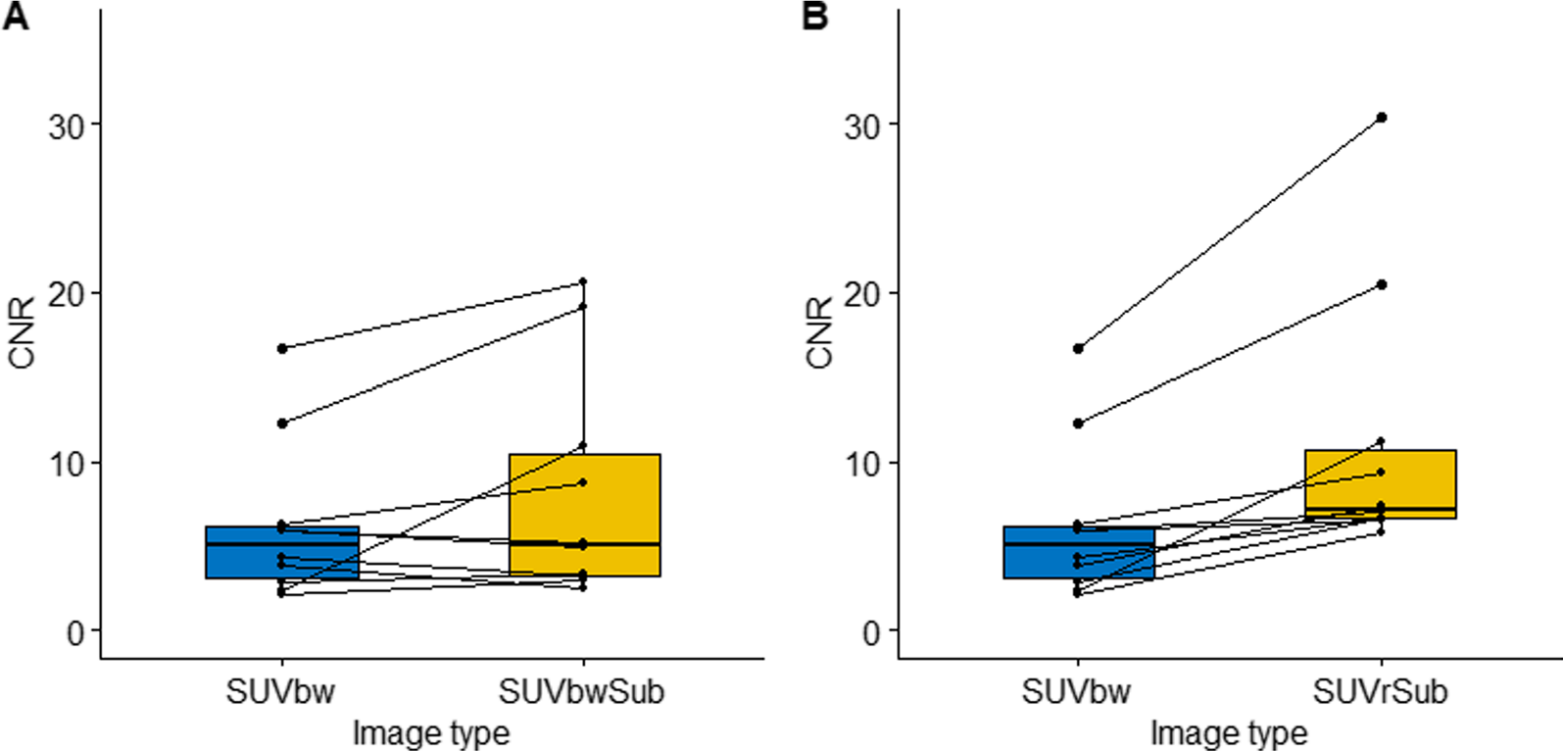
Contrast to noise for original Met-PET compared with both subtraction images. Linked box plots show the CNR using the SUVbw (original) images compared with the subtraction images generated from **a** the two SUVbw images and **b** the two SUVr [cerebellum] images

Both subtraction imaging techniques and the standard clinical Met-PET scans were compared using CNR (see Eq. 1 and Fig. 5) for ten patients with a pituitary tumor that had confirmed lateralization. All three image types for all ten patients correctly lateralized to the known location of the tumor with mean CNR of 6.2, 8.1 and 11.1 for SUVbw, SUVbwSub and SUVrSub [cerebellum], respectively. Therefore on average, the SUVrSub [cerebellum] had the highest CNR with the SUVbwSub also improving detectability over SUVbw. However, although SUVrSub [cerebellum] improved detectability in all ten patients, SUVbwSub only improved this in six subjects. Therefore in four patients, the detectability was higher for SUVbw than SUVbwSub. Figure 6 shows paired CNR measurements for both subtraction techniques compared with the original SUVbw image.

**Fig. 7:**
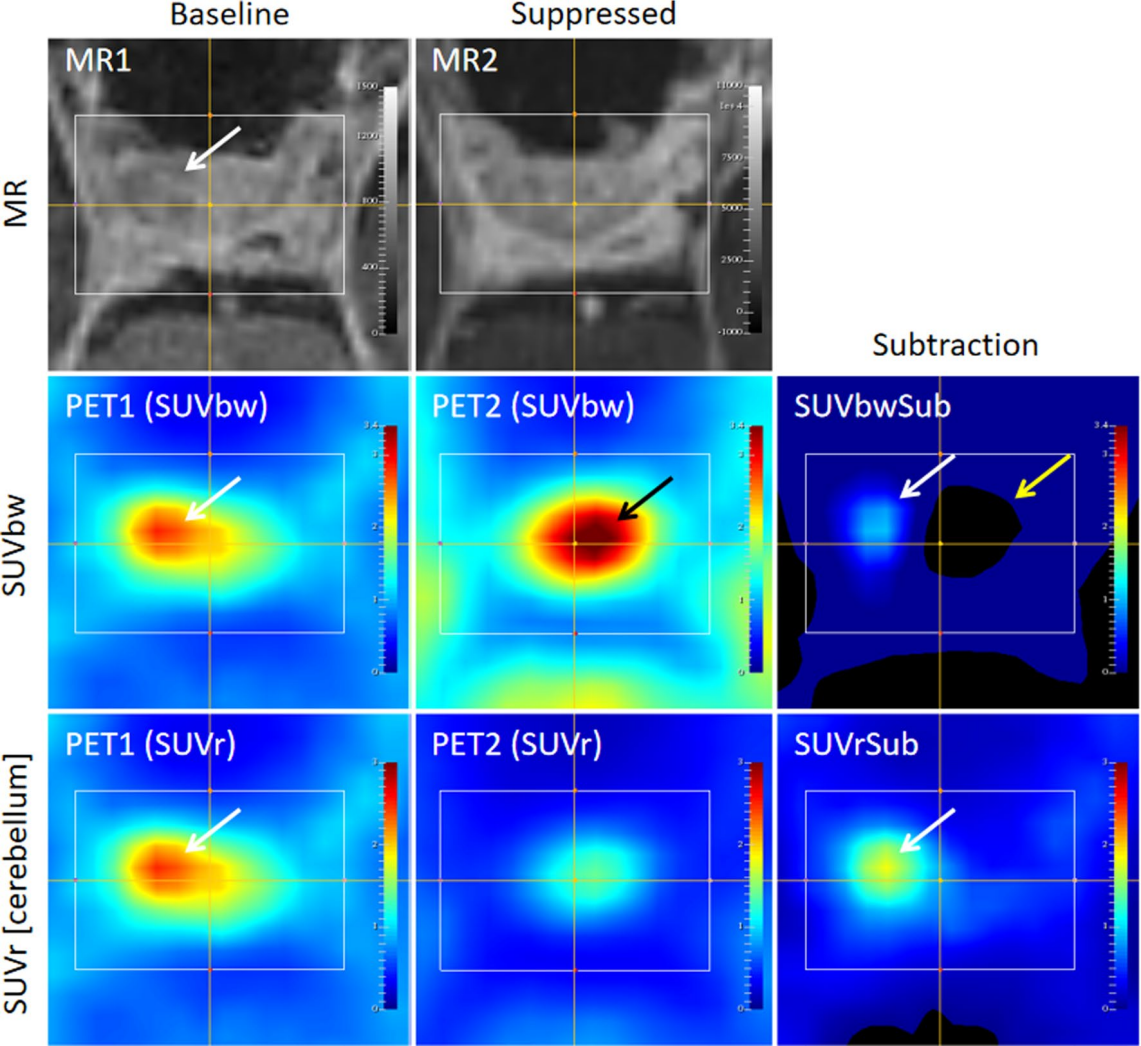
Findings in subject 4. The white arrows highlight the site of the suspected pituitary tumor on the baseline MR (MR1), the skewed tracer distribution of the baseline Met-PET (PET1) and the focal signal seen on the subtraction images. The black arrow highlights the variability of the Met-PET signal, and the yellow arrow highlights the effect of this on the subtraction image; the SUVbw values of the suppressed Met-PET are higher than those of the baseline, and therefore, the difference between them (seen in the SUVbwSub image) is not as pronounced as that of the normalized images (seen in the SUVrSub [cerebellum] image)

The images for a representative subject are shown in Fig. 7. This example is one of the subjects where the CNR was highest for the SUVrSub [cerebellum] and lowest for the SUVbwSub. The images highlight that the subtraction image generated from the SUVbw images is less pronounced than the normalized PET images because the SUVbw values seen in the suppressed scan are globally higher than those of the baseline scan. The inconsistency in uptake between the scans is improved by normalization, which is why the SUVrSub [cerebellum] image is much more pronounced.

## Discussion

We have shown that subtraction imaging, generated from normalized Met-PET scans performed before and after medical suppression of tumor function, permits accurate lateralization of pituitary tumors, with an improved CNR when compared with standard clinical imaging.

The registration process was optimized for direct registration between the PET and MRI images (Fig. 1), without the need for a hybrid CT. This reduced the computational time required for the process and, more importantly, negates the need for additional registrations when there are small misalignments between the PET and the hybrid CT. These misregistrations happen infrequently and are easily seen by expert reviewers in standard clinical practice due to physiological uptake in surrounding structures (e.g., gray matter, bone marrow). However, these misalignments would not be as easily seen in the subtraction image, because much of the surrounding uptake is suppressed and therefore direct registration between PET and MRI is considered an important part of the overall process.

The process of creating subtraction images (Fig. 1, step 4) required additional registration between PET2 and PET1 because although the steps of the initial registration (Fig. 1, steps 1–3) result in the four images being aligned, the subtraction process will highlight any small differences caused by minor misalignments. This additional registration typically took less time (< 10 s) than the other registration steps (1–3) because both images are smaller than the MRI images.

The PET2 to PET1 registration was also used to resample the PET2 image into an identical matrix as PET1 (Fig. 1). These identical matrices are crucial to be able to perform voxel-wise calculations. Attempts to create the subtraction images by using the world coordinate system were not as successful because the same interpolation of the voxel values was still required but had to be performed as part of the subtraction process and added unnecessary computational complexity.

The process of normalization involved outlining the reference region using a semi-automated (using a thresholding tool) method. Therefore, outlining these regions could be improved by automating the process using artificial intelligence (AI). There is a growing body of work that shows that AI and in particular deep-learning techniques (DL) have potential in automatically segmenting various areas of the body including the cerebellum [26].

To assess which reference region would be used for normalization, we compared the maximum signal in the pituitary gland using two images from each of the healthy volunteers acquired at different time points. As well as the regions we also compared the differences seen in the original SUVbw images in case no reference region was needed. We found that the maximum signal from the SUVbw images could vary on average by more than 20% (see Fig. 5). Differences of this magnitude are critical when creating images to highlight differences between pairs of datasets, but are arguably even more important in clinical practice when a patient is returning for surveillance imaging. Therefore, it could be recommended that all Met-PET images are normalized using a reference ROI before reading, although more data would be needed to confirm our findings. Using the six healthy volunteers, our results indicate that all three reference regions (cerebellum, gray matter and pons) reduce the variation in maximum pituitary gland signal when compared to non-normalized SUVbw images (Fig. 6). Using the cerebellum led to the lowest variations and consequently was selected when creating the subtraction images from normalized datasets. These normalized datasets were compared with the original (non-normalized) datasets to find the technique that resulted in the highest detectability of the tumor (as defined by Eq. 1).

Quantifying the CNR between the left and right side of the pituitary gland (Fig. 4) acted as a good surrogate for lateralization, correctly locating the tumor side in all cases for all three image types (SUVbw, SUVbwSub and SUVrSub [cerebellum]). Although it performed well, it was still limited because it only considered the pixel with maximum intensity from each side. Other metrics, such as the mean signal and absolute signal on each side, were ineffective in comparing the subtraction imaging techniques because they did not account for the noise in the image. Since the noise in the images varied and was not comparable between techniques, taking into account this noise was especially important when comparing those techniques that had very high or very low signal. Only by comparing the contrast between the PET signal and noise, we were able to analyze the techniques in a meaningful way.

The CNR results indicate that normalized subtraction imaging (SUVrSub [cerebellum]) improved detectability of pituitary adenomas when directly compared to the original (SUVbw) images and non-normalized (SUVbwSub) images. In every case, the SUVrSub [cerebellum] image had the highest CNR out of the three image sets, but importantly the SUVbwSub images did not always generate a higher CNR over SUVbw images. In four out of the ten patients, the CNR was lower after subtraction which highlights the importance of normalization.

This technique could also find application with other pituitary tumor subtypes where there is the possibility to suppress tumor function using medical therapy (for example, prolactinoma scanned pre- and post-dopamine agonist therapy; acromegaly scanned pre- and post-somatostatin receptor ligand). Similarly, the basic principles underpinning our methodology are potentially applicable to other PET ligands and other tumors. In particular, PET ligands that target tumoral SSTR receptor expression in TSH-secreting pituitary adenomas may be considered [27] although the use of SRL therapy to suppress PET uptake may not yield the same results seen in this study with Met-PET because the ‘cold’ ligand will directly compete with the PET ligand for somatostatin receptor binding sites.

## Conclusion

In conclusion, this study demonstrates that pituitary adenoma localization can be aided by normalized subtraction imaging. By highlighting areas of change between a baseline Met-PET image and registered suppressed Met-PET, this novel technique can allow improved localization of an adenoma when compared with conventional imaging modalities, especially when the cerebellum is used as a reference region. Implementation of this technique in clinical practice may be of particular added value when definitive intervention (e.g., transsphenoidal pituitary surgery or stereotactic radiosurgery) is being considered, to guide targeted intervention and mitigate the risk of disrupting normal pituitary gland function.

## Data Availability

The datasets used and/or analyzed during the current study are available from the corresponding author on reasonable request.

## Abbreviations

AC: Attenuation correction
AI: Artificial intelligence
CoV: Coefficient of variation
CNR: Contrast-to-noise ratio
Co-Reg: Co-registration of two or more images
CT: X-ray computed tomography
FSPGR: Type of MRI imaging—fast spoiled gradient (recalled) echo
Met-PET: ^11^C-methionine PET
MRI: Magnetic resonance imaging
OSEM: Ordered subset expectation maximization
PA: Pituitary adenoma
PET: Positron emission tomography
SRL: Somatostatin receptor ligand
SUV: Standardized uptake value
SUVbw: Standardized uptake value image using body weight
SUVr: Standardized uptake value ratio
SUVbwSub: Subtraction image generated from SUVbw images
SUVrSub: Subtraction image generated from SUVr images
TE: Echo time used in MRI
TOF: Time of flight
TR: Repetition time used in MRI
TSS: Transsphenoidal pituitary surgery
VOI: Volume of interest
